# Nutritional Modulation of Insulin Resistance

**DOI:** 10.6064/2012/424780

**Published:** 2012-09-05

**Authors:** Martin O. Weickert

**Affiliations:** ^1^Warwickshire Institute for the Study of Diabetes, Endocrinology and Metabolism, University Hospitals Coventry and Warwickshire NHS Trust, Coventry CV2 2DX, UK; ^2^Division of Metabolic and Vascular Health, Warwick Medical School, The University of Warwick, Coventry CV4 7AL, UK

## Abstract

Insulin resistance has been proposed as the strongest single predictor for the development of Type 2 Diabetes (T2DM). Chronic oversupply of energy from food, together with inadequate physical activity, have been recognized as the most relevant factors leading to overweight, abdominal adiposity, insulin resistance, and finally T2DM. Conversely, energy reduced diets almost invariably to facilitate weight loss and reduce abdominal fat mass and insulin resistance. However, sustained weight loss is generally difficult to achieve, and distinct metabolic characteristics in patients with T2DM further compromise success. Therefore, investigating the effects of modulating the macronutrient composition of isoenergetic diets is an interesting concept that may lead to additional important insights. Metabolic effects of various different dietary concepts and strategies have been claimed, but results from randomized controlled studies and particularly from longer-term-controlled interventions in humans are often lacking. However, some of these concepts are supported by recent research, at least in animal models and short-term studies in humans. This paper provides an update of the current literature regarding the role of nutrition in the modulation of insulin resistance, which includes the discussion of weight-loss-independent metabolic effects of commonly used dietary concepts.

## 1. Introduction

Western diseases are epidemic following major changes in lifestyle including physical activity, and the replacement of the traditional high-fiber diet by a diet rich in fat, sugar and protein at the beginning of the 20th century [1–6].

One in three Americans born in 2000 or later and some 50% of members of high-risk ethnic populations are expected to develop type 2 diabetes (T2DM) [7], with all its known negative consequences including renal failure, cardiovascular disease, blindness, neuropathy, amputations, arthritis, obstructive sleep apnoea syndrome, psychological ill health, and premature mortality [8]. It is undisputed that chronic overconsumption of energy in the absence of adequate physical activity leads to weight gain and excess intraabdominal fat, factors that strongly predispose to insulin resistance [9], and finally the development of T2DM [10, 11]. Appropriate dietary measures as part of a healthy lifestyle are known to substantially reduce these risks. Given that loss of excess body weight and reduction of the intraabdominal fat mass are strongly linked with improved insulin sensitivity [9], any energy reduced and safe diet that can be sustained in the long term may be used for both prevention and treatment of insulin resistance, particularly in high-risk subjects. However, sustained weight loss with any dietary strategy is difficult to achieve. Therefore, apart from weight loss and reduction of abdominal fat mass, lifestyle measures that are aimed to improve or prevent insulin sensitivity independent of weight loss are of interest. Evidence is increasing that isoenergetic changes in the quality of ingested foods and in the macronutrient composition of the diet appear to exert additional important effects on insulin sensitivity [12–15], with protective, neutral, or adverse effects of specific foods [6, 16–18]. However, most of the available data so far is derived from *in vitro* and animal studies, epidemiological studies that do not allow commenting on causality, or from relatively small and short-term intervention studies in humans. Therefore, many of the currently proposed beneficial dietary strategies remain controversial both regarding their safety and efficiency for preventing insulin resistance and T2DM in the long term.

This paper reviews current concepts and controversies regarding the modulation of insulin resistance, glucose metabolism, and diabetes risk using dietary measures.

## 2. Methods

This is a narrative review. PubMed was searched for original papers and review articles up to July 2012, with a combination of query terms that included “insulin resistance,” “type 2 diabetes,” “diet,” “nutrition,” “metabolic syndrome,” “dyslipidemia,” “adipokines,” “gut hormones,” “pro-inflammatory factors,” “obesity,” and many others that were assumed to be relevant. Relevant articles were further selected among references in published papers. Using these search criteria, more than 1000 relevant original papers and review articles were identified. Studies and review articles that were assumed to be relevant for covering the respective areas were then hand selected, and articles that did not report substantially different outcomes as compared with the selected ones were excluded. When data from larger trials were available, studies with small sample sizes were excluded, as well as studies that did not report results from a control group, and studies that showed high losses to followup and/or differential losses between the comparison groups.

## 3. Recommendations in Current Guidelines

Nutritional recommendations for the treatment of patients with T2DM and subjects at high risk of developing diabetes [19] generally recommend weight loss of at least 7% in overweight/obese patients; restriction of the intake of saturated fats to <7% of energy intake; a cholesterol intake <200 mg/day; restriction of *trans *fat intake; a high-fiber intake of at least 14 g/1000 kcal; in newer guidelines, lifted restrictions of protein intake, for example, protein intake of 15–20% of energy as long as kidney function is normal [19]. It is also assumed that the use of low glycemic index (GI) and glycemic load (GL) carbohydrates may provide a modest additional benefit for glycemic control over that observed when total carbohydrate is considered alone [19], and, because of lack of evidence of efficacy and increasing concerns related to long-term safety, discouragement of routine supplementation with antioxidants, such as vitamins E, C, and carotene [19, 20].

## 4. Effects of Weight Loss on Insulin Resistance and Diabetes Risk

Accumulation of intraabdominal fat mass is the most important cause of insulin resistance and T2DM. Simply being overweight (BMI > 25 kg/m^2^) raises the risk of developing T2DM by a factor of 3 [21]. It is known since decades that this effect can be effectively reversed by reduction of excess body weight [22]; in obese patients with poorly controlled T2DM even modest weight loss, if maintained, markedly reduces plasma glucose concentrations and improves markers of glucose metabolism [23–25]. Therefore, the recommendation to lose weight remains one of the key principles in the treatment of patients with T2DM [26].

However, even in the general overweight population sustained weight loss is difficult to achieve.Generally, in obese individuals energy expenditure begins to drop as soon as body weight starts to decline [27–29], and powerful hypothalamic hormonal responses are induced in an effort to maintain weight [27]. In addition to this, patients with diabetes appear to face further drawbacks for maintained success. Proposed factors include increased energy expenditure in the hyperglycemic state due to increased protein turnover that may drop toward normal after improvement of glycemic control [27, 30, 31] and reduced loss of the energy carrier glucose with the urine once glucose metabolism improves, resulting in retention of energy that may further contribute to weight regain if energy intake does not drop further [27]. Furthermore, many obese patients with T2DM are typically sedentary and may have relevant barriers to exercising, including neuropathy, foot ulcers, heart disease [27], and anxiousness to experience hypoglycemia. Another problem is medication with certain antidiabetic drugs that are known to cause weight gain such as insulin, sulfonylureas, and thiazolidinediones [27], further compromising the efforts to lose weight in these patients. Reflecting this, the typical weight loss trial in patients with T2DM either shows no relevant weight loss [32], or jojoing with an initially successful weight loss followed by a plateau after 4–6 months and subsequent weight regain [27]. Further complicating this issue is the observation fat mass is regained to a greater degree than is lean mass in those who do experience weight regain after initial weight loss [33]. As an example, when investigating the body composition in postmenopausal women after intentional weight loss, followed by weight regain, for every 1 kg fat lost during weight-loss intervention, 0.26 kg lean tissue was lost, but for every 1 kg fat regained over the following year, only 0.12 kg lean tissue was regained [33].

In addition, relevant exercise levels that would help to lose weight are difficult to achieve. Typically recommended exercise levels that are probably sufficient to improve glycemic control and cardiovascular risk (e.g., 150 min/week of brisk walking) are usually inefficient to achieve relevant weight loss [34]. The optimal volume of exercise to achieve sustained major weight loss appears to be much larger, requiring some 60 min/day or more when relying on exercise alone as a weight loss strategy [8, 34]. The combination of these factors likely contributes to the fact that aiming to achieve and maintain relevant weight loss with the recommendation of energy reduced diets and increased exercise levels often fails in overweight/obese patients with T2DM.

## 5. Effects of Diets Varying the Macronutrient Composition on Weight Loss

The only moderate effects of low fat diets on weight reduction [35] have led to a renaissance of various alternative dietary concepts, including food combining strategies, low-carbohydrate diets that are often high in dietary protein, high-fiber diets, and carbohydrate-rich diets that are aimed at modulating the postprandial glucose and insulin responses and as such the glycemic index (GI) of foods. Some of the most commonly used concepts are discussed in this paper.

### 5.1. Effects of Low-Fat Diets on Weight Loss

Short-term dietary intervention studies show that low-fat diets lead to weight loss in overweight individuals [36]. However, it is less clear whether a reduction in fat intake is more efficacious than other dietary restrictions in the long term. Fat-restricted diets appear to have no advantages compared with other calorie-restricted diets in achieving long-term weight loss in overweight or obese people. In some analyses participants lost slightly more weight on the control diets but this difference was small and not significantly different from the weight loss achieved through dietary fat restriction [37]. A major problem is poor adherence to low fat diets in the longer term, which appears to be particularly challenging in insulin-resistant subjects [38]. Low-carbohydrate, nonenergy-restricted diets appear to be at least as effective as low-fat, energy-restricted diets in inducing weight loss for up to 1 year [39].

### 5.2. Effects of High-Fiber Diets on Weight Loss

A high-fiber intake is emphasized in the recommendations of most nutritional and diabetes associations. Factors that are assumed to contribute to the beneficial effects of fiber intake include the bulking effect of adding low-energy food to the diet, and the slowing of gastric emptying and absorption of dietary carbohydrate and fat contents, a concept that is mainly attributed to viscous water soluble types of dietary fiber [15]. It is also assumed that dietary fiber intake increases satiety and beneficially influences efforts to lose weight [15]. Ludwig has shown that weight gain over a 10-year period correlated better with fiber intake than with the intake of dietary fat contents [40]. However, many other studies reported only moderate effects of fiber intake on weight loss, with no clear differences between the sort of fiber consumed [41], and data from published studies are in part inconclusive [35, 42]. Only few controlled studies investigated the effects of whole grain products on weight loss [43].

### 5.3. Effects of Low Glycemic Index (GI) Diets on Weight Loss

In a meta-analysis of six small studies (total number of participants *n* = 202) with short duration (5 weeks to 6 month), overweight or obese people on low glycemic index (GI) diets lost more weight and had better improvement in lipid profiles than those receiving other diets [44]. Furthermore, in studies comparing low-GI diets with conventional-restricted energy low-fat diets, participants fared at least on the low-GI diets as well, even though total energy intake was *ad libitum* [44]. However, other studies have shown no advantage of a low versus a high GI diets regarding weight loss [45]. In one of the largest intervention studies published to date, weight regain at 1 year was only marginally lower with a reduction of the GI [46]. Generally, the small number of participants and the relative short duration of the available studies do not allow final conclusions regarding the effect of low-GI diets as a weight loss instrument.

### 5.4. Effects of Low-Carbohydrate High-Protein Diets on Weight Loss

Energy-reduced diets are difficult to follow because they often require elimination of certain foods, leading to poor adherence and limited success. Increasing the protein content of the diet has been shown to be more successful in achieving weight loss, in comparison to low fat diets, at least in the short term [35, 47]. The most common concept includes carbohydrate restriction and increasing the intake of dietary protein. It is not entirely clear whether low-carbohydrate diets work mainly *via* increasing the protein content of the diet, or whether reduced carbohydrate intake *per se*, increased fat intake, or a combination of these factors are the key principles. However, some studies indicate that increasing the protein content of the diet alone, without restricting carbohydrate intake, leads to significantly reduced appetite and energy intake, followed by weight loss and reduction of fat mass, although the small sample size of the study (*n* = 19), the relatively short duration (12 weeks), and the lack of a control group need to be mentioned [48].

Very high-protein diets (protein > 30–35% of energy) with a reduction in both dietary carbohydrate and fat content have been also proposed, but these are difficult to achieve and maintain in daily life without the use of dietary supplements. The same is true for very low-carbohydrate diets, with poor adherence rates even under controlled conditions in dietary intervention studies [49, 50]. More importantly, there are reports indicating that extreme changes in the diet such as very low-carbohydrate diets may have serious adverse effects on health [51].

Moderately low-carbohydrate high-protein diets are attractive because they promise rapid weight loss without having to count calories, and the consumption of many palatable foods is not restricted [35]. These diets also appear to have beneficial effects on blood lipids, body composition, and weight loss, at least in the short term [47]. Better weight loss with low-carbohydrate diets may be explained by higher satiating properties of dietary protein in comparison to other macronutrients, an effect that has been shown both in short- [52–56] and long-term [57, 58] studies. Further factors involved may include a reduced variety of allowed foods, and an aversion against dietary fat content in the absence of relevant amounts of carbohydrates [59], whereas the often proposed ketosis is less likely to play a key role in this context [60, 61]. Lowering the percent protein of the diet from 15% to 10% results in higher total energy intake, predominantly from savoury-flavoured foods available between meals [62], reiterating that a higher dietary protein intake may help to reduce energy intake. High-protein intake may further lead to increased thermogenesis, potentially further contributing to favorable effects of these diets on the regulation of body weight [63–65], and there is evidence that high-protein intake reduces fat mass and increases lean mass in overweight and obese subjects [66].

However, long-term adherence to any diet is a key factor for maintained weight loss [67], with many studies indicating that initially successful high-protein intake is often not sustained in the longer term, even in the setting of controlled dietary interventions [49, 50, 68]. Therefore, relevant long-term adherence to these diets with sustained weight loss may be difficult to achieve. More importantly, evidence is accumulating casting doubt on the long-term safety of these diets, with novel data from longer term prospective cohort studies indicating potential adverse effects on both the risk of developing T2DM [69] and cardiovascular risk factors [70].

### 5.5. Effects of Food Combining Strategies

Concepts such as food combining are largely proposed in the lay press, but scientific evidence supporting that these are superior to any other energy restricted diet is lacking [71–73]. In a 6-week study investigating energy reduced but isoenergetic food combining diets with balanced diets with comparable macronutrient composition in 54 obese subjects, authors observed no differences in weight loss between groups, but a tendency to less pronounced weight loss and reduction in body fat mass was noted in the food combining group [73]. However, only few controlled studies have compared these concepts.

### 5.6. Importance of Adherence to a Specific Diet

Various different dietary concepts have been proposed to improve the effects of dietary strategies on weight loss and its metabolic consequences. Interestingly, more important than the choice of a specific diet appears to be the adherence to and, related to this, the long-term sustainability of the chosen strategy. In a study investigating the effectiveness of four currently widely used diets (Weight Watchers, Zone, Atkins, or Ornish) on weight loss, each diet resulted in modestly reduced body weight and improvement of several cardiac risk factors at 1 year. However, overall adherence rates to all four diets was low, whereas increased adherence was associated with significantly greater weight loss and cardiac risk factor reductions for each diet [67]. In agreement with this, energy-reduced diets resulted in clinically meaningful weight loss regardless of the macronutrient composition (fat, protein or carbohydrates) in a 2-year study in 811 overweight adults. All diets showed comparable effects on feelings of satiety and hunger, satisfaction with the diet, and attendance rates at group sessions, whereas attendance of instructional sessions was strongly associated with weight loss (0.2 kg per session attended), further indicating that adherence to any chosen diet may be a crucial factor and may be more relevant than the macronutrient composition of the diet *per se* [74].

## 6. Dietary Concepts Using Modulation of Macronutrient Composition without Energy Restriction

**Figure 1:**
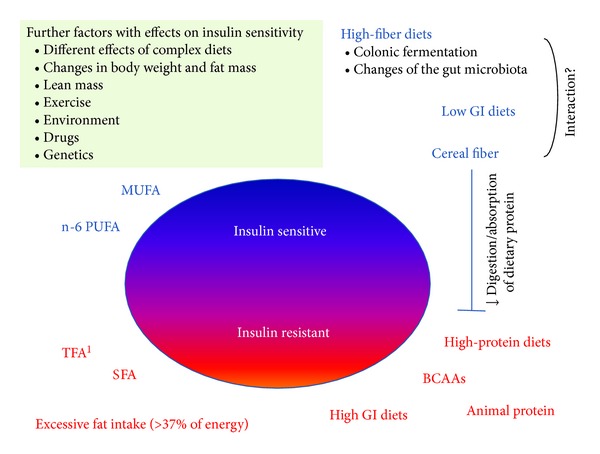
Dietary concepts using modulation of macronutrient composition without energy restriction. There appear to be relevant interspecies differences when comparing metabolic effects of specific fatty acids. For example, in humans, n-6 PUFA may improve insulin resistance and diabetes risk, whereas n-3 PUFA from marine origin improve insulin sensitivity in rodent models but not in humans. No long-term-randomized trials have been published to date that investigated the effect of dietary fat composition on diabetes risk. High-fiber diets and particularly diets high in insoluble cereal fiber appear to improve whole-body insulin sensitivity, possibly by interference with the digestion and/or absorption of dietary protein and as such preventing the amino-acid-induced activation of the mTOR/S6K1 signalling pathway. Separating the effects of high-fiber diets from potentially independent effects of diets varying in the glycemic index (GI) is challenging. In rodents, changes in the composition of the gut microbiota and colonic fermentation with the production of short chain fatty acids (SCFA) appear to be involved, but it remains to be shown whether this applies also in humans. Adverse effects of high-protein diets on insulin sensitivity may be partly compensated by satiating effects of dietary protein and consequent weight loss, and increases in lean mass, but long-term maintenance of weight loss with any diet appears to be difficult to achieve. MUFA, monounsaturated fatty acids; PUFA, polyunsaturated fatty acids; TFA, *trans *unsaturated fatty acids; SFA, saturated fatty acids; GI, glycemic index; BCAA, branched chain amino acids.

Although weight loss and reduction of abdominal fat mass in patients with T2DM are powerful tools for reducing insulin resistance in principle, sustained relevant weight loss in these patients appears to be difficult to achieve. An increasing number of studies indicate that isoenergetic changes in the macronutrient composition and the quality of ingested foods may exert additional important effects on insulin sensitivity, independent of weight loss. Therefore, it seems reasonable to explore specific metabolic effects of different (isoenergetic) foods and macronutrients on insulin sensitivity both in patients with T2DM and in individuals who are at high risk of developing T2DM [12–15]. Some of the potentially involved concepts and controversies are depicted in Figure 1.

### 6.1. Metabolic Effects of the Modulation of Dietary Fat Contents

An excessive intake of total fat (>37% of daily energy intake) reduces insulin sensitivity irrespective of the composition of fatty acids (FA) in the diet [14]. Involved factors, apart from excessive energy intake and weight gain, may include impaired glucose transport, decreased binding of insulin to its receptors, and accumulation of stored triacylglycerols in skeletal muscle [14, 75–77]. Therefore, reducing the intake of excess fat from a diet is assumed to be beneficial. However, many overweight/obese patients have difficulties adhering to these diets particularly in the longer term, resulting in only limited success.

Although not fat-restricted, Mediterranean style diets exert relevant beneficial effects on insulin resistance, diabetes risk, and cardiovascular health [12, 13], indicating that the type and composition of dietary fat are likely to be important. Especially under conditions of a more moderate fat intake (<30%) different types of dietary fat appear to have a relevant role in the modulation of diet-induced insulin resistance [78]. Dietary fat is a heterogeneous mixture of different FA, with monounsaturated (MUFA), polyunsaturated (PUFA), saturated fatty acids (SFA), and *trans* unsaturated fatty acids (TFA) as the main components [14]. The adverse effects of TFA on cardiovascular disease are well established but their role in the development of IR and T2DM is less clear [13]. A high intake of TFA may lead to insulin resistance and show adverse effects on cardiovascular disease [13, 79, 80]. Studies in rats have shown that TFA induces insulin resistance both when compared with low fat diets [81] and diets rich in SFA [82, 83]. Moreover, adverse effects of TFA intake on insulin sensitivity may be greater in individuals more predisposed to insulin resistance [84]. In the Nurses' Health Study, a dose-dependent association between TFA intake and risk of T2DM was shown [79], probably related to a TFA-induced increase in inflammatory cytokines [13].

Apart from TFA, many bakery products and high-energy prepacked foods contain also relevant amounts of SFA that may be sufficient to increase insulin resistance and risk of diabetes [14, 85]. Epidemiological studies indicate a direct relation of dietary SFA with the incidence of insulin resistance or T2DM [86, 87], whereas replacing SFA by MUFA may improve insulin sensitivity [78] and beneficially influence blood pressure, low-density lipoprotein (LDL) cholesterol, and triacylglycerols [88]. However, recent research also indicates that specific SFA largely differ in function, structure, and metabolic effects, with some SFA having important and specific biological roles [89].

SFA, under conditions of hyperglycemia, can exert damaging effects on **β** cells, a concept known as glucolipotoxicity [90–93]. Apart from influencing key enzyme activities and transcription factors on an intracellular level [89], a SFA-mediated increase in intramyocellular lipid content and composition may also activate specific serine kinases, finally leading to insulin resistance [94, 95]. Interestingly, FA-induced endoplasmic reticulum stress leading to the activation of sterol-regulatory element-binding protein-1 (SREBP-1) [96] may link both high-fat diet-induced obesity with insulin resistance, and insulin resistance and loss of **β**-cells on a molecular level. Finally, SFA may influence inflammatory pathways which are related to impaired insulin sensitivity [97]. However, treatment with high-dose acetylic salicylic acid has been shown to reverse lipid-induced insulin resistance in humans, although no changes in selected inflammatory markers were detected [98].

The underlying mechanisms involved in MUFA-induced improvement of insulin sensitivity are subject to further research but may involve effects on cell membrane FA composition [13], with functional effects on membrane fluidity, ion permeability, insulin receptor binding/affinity [13], and upregulation of glucose transporters [14, 99]. Other involved mechanisms might be related to alterations in incretin responses [14, 100, 101] and cytoprotective effects on beta-cell function [14, 102, 103]. In insulin resistant subjects, an isoenergetic MUFA-rich diet prevented central fat redistribution and insulin resistance induced by a carbohydrate-rich diet [104]. Despite these findings, an increased intake of MUFA was not associated with reduced risk of T2DM in prospective cohort studies [105].

PUFA also may reduce insulin resistance based on their anti-inflammatory properties, probably mediated through effects on toll-like receptors (TLRs) [14]. Contrary to the proinflammatory profile of SFA, n-3, PUFA have been shown to inhibit TLR-2 and TLR-4 [106]. Further postulated effects of PUFA on insulin action may include beneficial alterations in membrane fluidity, increased binding affinity of the insulin receptor, and improved glucose transport into cells *via* glucose transporters [107–109], as well as effects on circulating triglycerides and low-density lipoprotein particles [110]. Finally, effects on the regulation of various genes that are involved in lipid and carbohydrate metabolism have been shown which include peroxisome proliferator-activated receptors, SREBP-1c, hepatic nuclear factors, retinoid X receptors, and liver X receptors [14, 111].

It is, however, important to note that no long-term-randomized trials have been published to date that investigated the effect of dietary fat composition on diabetes risk. Furthermore, many studies investigating potential mechanisms for FA-induced changes in insulin sensitivity have been performed *in vitro* or in animal models only. There appear to be relevant interspecies differences when comparing metabolic effects of specific FA. As an example, in humans, n-6 PUFA may improve insulin resistance and diabetes risk [13], whereas the reported beneficial effects of n-3 PUFA consumption from marine origin, as shown in rodent models, do not appear to apply [9, 13, 77]. Therefore, further research is needed to investigate the effects of modulation of FA composition on insulin resistance and diabetes risk in humans.

### 6.2. Potential Effects of the Genetic Background

Genetic differences between subjects exposed to a lipid challenge may play an additional role. We had recently hypothesized that amino acid replacements in liver fatty acid binding protein (L-FABP) might alter its function and thereby affect glucose metabolism in lipid-exposed subjects, as indicated by studies in L-FABP knockout mice [112]. Endogenous glucose production (EGP), gluconeogenesis, and glycogenolysis were measured in healthy carriers of the only common Thr(94)-to-Ala amino acid replacement (Ala/Ala(94)) versus age-, sex-, BMI-, and waist-matched wild-type (Thr/Thr(94)) controls at baseline and after 320 min lipid/heparin-somatostatin-insulin-glucagon clamps. Whole-body glucose disposal was further investigated in a subset, using euglycemic-hyperinsulinemic clamps without and with lipid/heparin infusion. The common Ala/Ala(94)-mutation contributed significantly to reduced glycogenolysis and less severe hyperglycemia in lipid-exposed humans and was further associated with reduced body weight in a large cohort [113]. Whole-body glucose disposal was not different between lipid-exposed L-FABP genotypes [113]. Importantly, investigation of L-FABP phenotypes in the basal overnight-fasted state yielded incomplete information, and a challenge test was essential to detect phenotypical differences in glucose metabolism between L-FABP genotypes [113]. Results indicated that L-FABP may not play a significant role in a normal diet but may contribute to disturbed glucose metabolism in a high-fat diet. However, various other factors unrelated to the L-FABP genotype are difficult to control for in human studies and may have influenced the results. Furthermore, results were obtained under highly experimental conditions using somatostatin infusion and replacement of insulin and glucagon in low fasting doses which may be well different from the situation after the intake of a high fat meal. There could be also ethical implications, particularly when investigating a SNP with potentially adverse effects on health, since even under blinded conditions the likelihood of a participant having the adverse mutation increases to 50% solely by inclusion in the study. Given these difficulties, very few human studies have been performed to date using a similar approach. Future research will be needed to further investigate the influence of the genetic background on diet induced-insulin resistance, with the final aim to design personalised tailored diets that are individually adapted to the needs and metabolic responses of the respective subject.

### 6.3. Metabolic Effects of Low-Carbohydrate High-Protein Diets

Reduction of carbohydrate content of a diet can be achieved by increasing the content of dietary protein, fat, or a combination of both at the expense of dietary carbohydrates. Since an excess intake of dietary fat is assumed to have unfavorable effects on health, many popular low-carbohydrate diets suggest increasing the content of dietary protein. Apart from better weight loss, these diets appear to have some advantages over other diets that include beneficial effects on body fat distribution, blood pressure, and HDL cholesterol [47, 66].

However, recent studies also indicate that high-protein diets could have detrimental effects on health in the longer term [114, 115]. Wang and colleagues have investigated the metabolite profiles in 2.422 normoglycemic individuals who were followed for 12 years. Of these participants, 201 developed diabetes [116]. Five branched chain and aromatic amino acids (isoleucine, leucine, valine, tyrosine, and phenylalanine) showed significant associations with future diabetes, and results were replicated in an independent, prospective cohort. [116]. Authors proposed amino acid profiling as a potential predictor for future diabetes, but a potential causal link between dietary protein intake and future diabetes cannot be excluded. In fact, there is increasing evidence that longer term high-protein intake may have detrimental effects on insulin resistance [68, 117–123], diabetes risk [69], and the risk of developing cardiovascular disease [70].

Therefore, the long-term safety of high-protein diets remains to be investigated [46, 69, 70]. In the Diet, Obesity and Genes (DiOGenes) trial weight regain at 1 year was only marginally lower with a higher protein intake [46]. Insulin sensitivity was not measured in DiOGenes but both the high-protein and the high GI diets significantly increased markers of low-grade inflammation [124], which could result in worsening of insulin resistance. Indeed, a recent study from our group showed significant and clinically relevant worsening of insulin sensitivity with an isoenergetic plant-based high-protein diet, as measured using euglycemic hyperinsulinemic clamps and stable isotope methods [68]. This negative effect on insulin sensitivity was observed despite tailoring the amino acid profile in the high-protein diet to a composition with assumed beneficial metabolic effects [68]. Furthermore, healthy humans that are exposed to amino acid infusions rapidly develop insulin resistance [120], with inhibition of glucose uptake being driven through phosphorylation of downstream factors of the insulin signaling cascade by translation initiation factor serine-kinase-6-1 (S6K1) [120, 121]. In agreement with this, longer term high-protein intake has been shown to result in whole-body insulin resistance [68, 118], associated with upregulation of factors involved in the mammalian target of rapamycin (mTOR)/S6K1 signalling pathway [68], increased stimulation of glucagon and insulin within the endocrine pancreas, high glycogen turnover [118] and stimulation of gluconeogenesis [68, 118].

In the short term, these negative effects of dietary protein on insulin sensitivity may be compensated by high-protein diet-induced weight loss, and, at least in physically active people, relevant increases in lean mass that are also mediated via the mTOR/S6K1 pathway [121]. However, most subjects on weight loss diets are overweight/obese and typically sedentary. Relevant increases in lean mass are unlikely to be achieved under such conditions. In further agreement that high-protein diets may deteriorate glucose metabolism, it was recently shown in a large prospective cohort with 10 years followup that consuming 5% of energy from both animal and total protein at the expense of carbohydrates or fat increases diabetes risk by as much as 30% [69]. This reinforces the theory that high-protein diets can have adverse effects on glucose metabolism. Another recent study showed that low-carbohydrate high-protein diets, used on a regular basis and without consideration of the nature of carbohydrates or the source of proteins, are also associated with increased risk of cardiovascular disease [70], thereby indicating a potential link between high-protein Western diets, T2DM, and cardiovascular risk.

The Carnivore Connection Hypothesis [125] proposes that during human evolution a scarcity of dietary carbohydrates together with high intake from animal proteins may have resulted in insulin resistance, thereby providing a survival and reproductive advantage by redirecting glucose from maternal use to fetal metabolism and increased birth weight and survival of the offspring [125]. However, such a diet could be deleterious in a high-carbohydrate environment [123]. In this context, it is interesting that populations who have only recently changed dietary habits from traditional high-protein hunter gatherer to modern high-carbohydrate diets show excessively high prevalence of insulin resistance and T2DM, as compared to European populations that switched to higher carbohydrate intake some 12,000 years ago [125, 126].

### 6.4. Effects of Modulating the Glycemic Index of Carbohydrate-Rich Foods

The glycemic index (GI) is a measure of the blood glucose-raising ability of available carbohydrates in foods and defined as the incremental area under the glycemic response curve (AUC) elicited by a portion of food containing 50 g available carbohydrate expressed as a percentage of the AUC elicited by 50 g glucose in the same subject [127]. Related is the concept of the glycemic load (GL), which takes account of the GI of a food and the amount eaten [128]. Low-GI and/or low-GL diets may reduce the risk of metabolic syndrome [129], T2DM [130, 131], cardiovascular disease [132], and chronic inflammation [133], probably by beneficial effects body weight [46, 134–136], insulin sensitivity [137, 138], *β*-cell function [139, 140], serum cholesterol [141, 142], glycemic control in diabetes [143]. In contrast, carbohydrates that are high in GI lead to rapid onset and pronounced increases of postprandial glucose and insulin concentrations that may compromise fat oxidation, fuel partitioning, and metabolic flexibility [16, 144]. High-GI diets have been linked to insulin resistance in epidemiological observations, whereas low-GI diets improved insulin sensitivity in patients with T2DM [16].

### 6.5. Separating Effects of the GI from Fiber Intake

However, separating the effects of single nutrients in complex foods on metabolic outcomes is not straight forward. Many low-GI diets are also rich in cereal fibers which are insoluble in water and have only negligible effects on the GI. However, cereal fiber intake is one of the strongest and most consistent independent factors associated with reduced risk of T2DM in prospective cohort studies [15, 17, 18, 145]. It cannot be excluded that at least some of the effects attributed to a low GI of carbohydrate-rich foods may be related to the cereal fiber content of the diet [15, 146]. Indeed, meta-analyses of large prospective cohort studies consistently show a 20–30% reduction of the risk of developing T2DM in subjects consuming diets high in cereal fiber (relative risk for extreme quintiles (RR); 0.67; 95% CI 0.62–0.72)) [18], whereas results regarding a protective effects of low-GI (and GL) foods are less conclusive [15].

### 6.6. Metabolic Effects of Dietary Fiber Intake

Apart from the mainly moderate weight loss that can be achieved with dietary fiber intake from most sources [41], further effects are likely involved in their beneficial action. Key-proposed principles include (i) improvement of total and LDL cholesterol levels that are mainly seen with the intake of viscous, soluble dietary fiber [147], whereas high-density lipoprotein (HDL) cholesterol and triacylglycerols are not relevantly changed [148]; (ii) the commonly proposed concept of fiber induced changes in the gut microbiota and fermentation of nondigestible fiber contents in the colon with increased production of short chain fatty acids (SCFA), with various metabolic effects that are assumed to be beneficial [15, 149–152].

However, and surprisingly, protective effects of fiber intake on the risk of developing insulin resistance and T2DM are consistently shown with a high intake of insoluble and only moderately fermentable cereal fibers and whole grains but not with a higher intake of fruit and vegetables that are generally richer in soluble, fermentable fiber contents [17, 18]. The main sources of cereal fiber in the large prospective cohort studies in the US are cellulose and hemicelluloses from wheat bran [15] that are insoluble in water, nonviscous and only moderately fermentable [15, 153]. Main sources of soluble, viscous, and fermentable fiber are typically fruit and vegetables [15]. The fact that neither intake of fruit (relative risk for extreme quintiles (RR) 0.96; 95% CI 0.88–1.04) nor vegetables (RR 1.04; 95% CI 0.94–1.15) shows any significant associations with reduced risk of developing T2DM in meta-analyses of large prospective cohort studies [18] does not support the hypothesis that viscous properties of dietary fiber influencing the GI or fiber-induced increases of SCFA are key driving factors for reduced diabetes risk, although additional potential beneficial metabolic effects are possible.

### 6.7. Fiber-Induced Changes in Colonic Fermentation and the Composition of the Gut Microbiota

Recent studies in both experimental models and humans show beneficial effects of food products with prebiotic properties on energy homeostasis, satiety regulation, and body weight gain [154], supporting the hypothesis that the composition of the gut microbiota may contribute to modulate metabolic processes associated with obesity, T2DM, and the metabolic syndrome [154]. However, these studies were almost invariably short term, and the role of such changes in long-term health benefits for the patient remains to be definitively proven [154].

In short-term studies in rodents, fiber-induced changes of the gut microbiota and increased production of SCFA in the colon appear to beneficially influence obesity and insulin resistance [155, 156]. Very few studies have investigated longer-term exposure to fermentable fiber in humans and animal models [152, 157, 158]. Track and colleagues investigated male Wistar rats over 67 weeks and found that short-term feeding with guar gum as compared with cellulose or bran had favorable effects on body weight and makers of carbohydrate tolerance. However, in the long term, these effects were absent, with a tendency to increased body weight and significantly higher pancreatic insulin and glucagon concentrations in the guar fed rats [158]. Similar results were observed in a long-term experiment from our group, comparing effects of adding different sorts of fiber to high fat diet fed C57BL/6J mice. Animals that were fed otherwise identical diets differing in soluble/fermentable (guar gum) versus insoluble/nonfermentable fiber (purified cereal fiber extract that was not fermentable *in vitro*) showed short-term beneficial effects of soluble fiber intake, but again these were completely abolished in the long term [157]: guar fed mice exhibited higher energy extraction of the diet with SCFA cumulatively contributing to total energy intake, resulting in a significantly more obese, insulin resistant phenotype when compared to mice receiving nonfermentable cereal fiber [157].

Such a phenomenon could be also relevant in humans, as indicated by early studies showing that fiber-induced increases of SCFA may contribute as much as 10% to total energy intake [159]. Interestingly, colonization of germ-free gnotobiotic mice with a prominent saccharolytic member of the normal human gut microbiota together with dominant human methane-producing germs results in markedly improved colonic fermentation and is associated with an obese phenotype in the host [160]. However, it is not clear whether these results apply in other species.

In humans, both the magnitude and the importance of fiber-induced changes in the gut microbiota and colonic fermentation remain largely unknown [155]. No long-term human studies exist that have investigated the effects of otherwise identical diets differing in soluble, fermentable versus insoluble cereal fiber contents only, and insight about potential beneficial effects of fiber-derived production of SCFA on insulin sensitivity is almost exclusively derived from short-term studies [161–164]. The same is true for studies that have focused on effects of prebiotics or carbohydrates with prebiotic properties in overweight persons or patients with T2DM, typically lasting between 4 and 12 weeks [165–172]. Only one long-term controlled study in 14 humans per group investigated the effects of an increase of the intake of moderately fermentable wheat fiber by 20 g/day on SCFA, glucagon, like peptide 1 (GLP-1) levels, and markers of insulin sensitivity over a 1-year period [152]. Authors showed that the wheat bran-rich diet increased circulating levels of GLP-1 at 12 month, but not earlier in the intervention, whereas plasma acetate and butyrate showed a transient increase at 9 month only [152]. Therefore, a convincing relation between fiber-induced changes in SCFA and circulating GLP-1 levels was not provided. Furthermore, it has been shown previously that propionate and butyrate are quantitatively taken up by the liver and can be almost undetectable in peripheral blood [173], whereas acetate might be not specifically attributed to SCFA production of gut microbiota. Portal vein blood sampling would yield more feasible results, but is too invasive for this purpose. No changes in markers of insulin sensitivity (homeostasis model assessment for insulin resistance (HOMA-IR)) were observed in the study of Freeland [152], but this does not exclude potential changes since other studies have reported fiber-induced improvement of insulin sensitivity using euglycemic hyperinsulinemic clamps as the gold standard method which would have been missed by using HOMA-IR only [68, 161].

Further evidence supporting that at least in humans fiber-induced increases in SCFA may not exclusively explain changes in insulin sensitivity comes from a series of recent studies. In a short-term-randomized-controlled cross-over study from our laboratory, 14 healthy participants consumed nonfermentable-purified cereal fiber extracts from wheat, or moderately fermentable extracts from oat hulls, or highly fermentable insoluble resistant starch, or low-fiber control. Both the consumption of the cereal fiber extracts and of resistant starch showed comparable and significant improvement of postprandial glucose handling in a second meal test the next day, although products largely differed in their fermentability as indicated by hydrogen breath tests [174]. These findings are supported by further studies showing similar second meal effects after the intake of various fiber-related substances largely differing in their rate of colonic fermentation [163, 175–179]. Notably, most of these studies were of too short duration to upregulate GLP-1 mRNA expression and showed not any effects on GLP-1 levels. Furthermore, in a 4-week study, Robertson et al. showed that consumption of highly fermentable resistant starch significantly improved whole-body insulin sensitivity using euglycemic hyperinsulinemic clamps, in the absence of any effect on circulating GLP-1 [162].

Despite the lack of convincing effects on colonic fermentation, insoluble cereal fiber intake, under isoenergetic conditions, increases whole-body insulin sensitivity in both short-term and more prolonged studies, as measured using euglycaemic-hyperinsulinaemic clamps [15, 68, 180]. These effects appear to be dose-dependent [15] but independent of colonic fermentation, changes in dominant groups of the gut microbiota, or circulating GLP-1 [15, 68, 153]. We have recently proposed a novel concept that could contribute to explaining improved insulin sensitivity with cereal fiber intake, showing that cereal fiber may hinder the digestion and/or absorption of dietary protein in the upper gut, thereby preventing amino-acid-induced activation of the mammalian target of rapamycin (mTOR)/translation initiation factor serine-kinase-6-1 (S6K1) signalling pathway that is known to drive insulin resistance [68, 120, 121]. Cereal diet-induced effects on whole-body insulin sensitivity were not matched by changes in markers of colonic fermentation and/or the composition of the gut microbiota, neither in the full model nor in additionally performed uncorrected subgroup analyses, and there was also no tendency to more pronounced effects after 18 versus 6 weeks of dietary intervention [153]. Insoluble cereal fibers generally show no major direct effects on the modulation of blood lipids, but may indirectly influence these parameters at the long term *via* improvement of whole body insulin sensitivity [15, 68, 161–163, 174, 175, 177, 178, 181]. Further potential effects of cereal fiber intake may include the modulation of gut hormones, adipokines, bile acid binding, and metabolite profiles which deserve further investigation.

## 7. Conclusions

Weight loss with the reduction of abdominal fat mass almost invariably reverses insulin resistance as a consequence of chronic excessive energy intake in relation to physical activity levels. Therefore, any safe and balanced life-style measures that lead to weight loss and can be sustained in the long term have the potential to improve insulin resistance and glycemic control. However, particularly in patients with T2DM, long-term-sustained weight loss appears to be difficult to achieve. In this situation, isoenergetic changes of the macronutrient composition and the quality of ingested foods may exert important additional effects on insulin sensitivity. Nutritional measures that could be useful in this context include a Mediterranean-like dietary pattern, but avoiding excess intake of dietary fat; substituting SFA and TFA by MUFA and n-6 PUFA; increasing cereal fiber intake, particularly when choosing a high-protein dietary strategy. Weight loss, the macronutrient composition of the respective diet, aerobic exercise, and resistance training all appear to improve insulin resistance, by distinct mechanisms. Therefore, a combination of these interventions tailored to the requirements of each subject should be one of the cornerstones of management [8, 19, 182]. For the planning of an optimal diet, further aspects are likely to be important which may include the consideration of gender differences [183], varying effects of specific diets depending on the ethnic background [184], genetic variation including potential differences in response to a diet in carriers of certain single-nucleotide polymorphisms, differences between individuals in the metabolite profiles, comorbidities, the intake and interactions of certain drugs, and the exposure to other environmental factors than the diet. Further, elucidating these aspects may ultimately lead to personalized dietary strategies that are tailored to the specific needs of the individual.
